# Commentary: Mind-Wandering Changes in Dysphoria

**DOI:** 10.3389/fpsyt.2021.722819

**Published:** 2021-07-14

**Authors:** Juergen Fell, Leila Chaieb

**Affiliations:** Department of Epileptology, University Hospital Bonn, Bonn, Germany

**Keywords:** mind wandering, mind blanking, experience sampling, tune-out, zone-out, SART, dysphoria

In this ambitious behavioral study, Guesdon and colleagues investigated mind wandering during two attentional tasks in dysphoric vs. non-dysphoric subjects. The first task was similar to a sustained attention to response task (SART) (1) commonly used to probe mind wandering. Digits were presented onscreen in random order and subjects had only to respond to the target digit with a button press. In the standard SART, subjects have to respond to the non-targets and to withhold their response for the targets. The second paradigm was an oddball task. Non-target slides with horizontal lines and rare target slides with vertical lines were presented and subjects were asked to respond to the targets (2). Moreover, a verbal statement with a negative or neutral/positive valence was shown at the center of each slide, which participants were instructed to ignore and which was intended to stimulate mind wandering toward the past. During each task nine thought probes were displayed (Supplementary Figure 2), which inquired whether subjects were “thinking about the task itself or something related to it” (on task, OT), whether they were “thinking about something unrelated to the task” (tune out, TO) or whether they “did not have any thought (mind was blank)” (zone out, ZO). Both “tune out” and “zone out” responses were interpreted as indicating off-task states, and were commonly addressed as mind wandering in the discussion. In the case of “tune out” responses distraction, meta-awareness, deliberateness, temporal orientation, specificity, perspective, and emotional valence were further probed.

As for major findings, the authors reported that the amount of off-task states (TO plus ZO) did not differ between groups. While the proportion of “tune out” responses was larger in dysphoric subjects, the proportion of “zone out” responses was larger in controls. Thought contents and characteristics did not significantly differ between groups. Regarding task performance, dysphoric subjects showed a higher variability of reaction times. Such an increased variability of reactions has been associated with mind wandering in prior studies (3, 4), and thus is consistent with the higher proportion of “tune out” responses in the dysphoric group. Among all participants, the numbers of response errors and “tune out” events were strongly correlated, which again is in accordance with previous investigations (5). In summary, the authors reported that the amount of on-task and off-task states is similar in both dysphoric and non-dysphoric subjects, but that off-task states with thoughts are more prevalent in dysphoria, and off-task states without thoughts are less prevalent.

This study, without a doubt, addresses a timely and clinically relevant topic. It is laudable, that the authors rely on experience sampling during attentional tasks to assess mind wandering, since this approach is much more reliable than evaluating self-rating scales. Unfortunately, the imprecise classification of mind wandering and mind blanking represents a severe methodological limitation. Although there are differing views (6), most experts in the field have used concepts which gravitate toward the following broad definitions: the term “mind wandering” refers to a state whereby attention is not focused on performance of the task at hand (7–10). The same goes for “mind blanking,” which is a state absent of thoughts, while mind wandering is a state with thoughts (11, 12).

Importantly, Guesdon and colleagues did not initially inquire whether subjects were focused on task performance or not, but deduced this from the thought probes themselves. Two methodological problems arise from this approach. First, thoughts “about the task itself or something related to it” (on task, OT) may have comprised mind wandering states. In case of “on task” responses, the authors further inquired (Supplementary Figure 2) whether subjects were “concentrating on the numbers/lines” or were “thinking about other aspects of the task (e.g., when will it finish? I am getting tired).” The first option is in line with the assumption that attention was focused on task performance. However, the second option obviously reflects a type of mind wandering, but appears to have been categorized as being “on task.”

Second, the authors classified “zone out” responses [“did not have any thought (mind was blank)”] as being “off task.” However, the absence of thoughts can actually mean that subjects were intensely focused on the tasks, since no thoughts were required for correct performance. In other words, it is not possible to differentiate between being “off task” or “on task” just by asking whether the subject experienced no thought. The authors half-heartedly address this problem in the discussion: “We cannot be certain whether “zone-out” events were mind wandering without meta-awareness rather than an absence of mind wandering.” Regrettably, this comment introduces an additional confusion, namely between meta-awareness and memory of mind wandering. Although both states are interrelated, it is possible that subjects are not aware of their mind wandering (i.e., absent meta-awareness), but remember it when prompted by a thought probe. Moreover, this comment ignores the possibility of genuine mind blanking.

As a result of these conceptual ambiguities, the authors' claims that dysphoric subjects show amounts of on-task and off-task states similar to controls, and have fewer off-task states without thoughts (or without memory of thoughts), remain in question. Alternatively, dysphoric subjects may actually have had fewer on-task and more off-task states, and may not have had less mind blanking than controls. The latter interpretation would be in line with findings from other studies [e.g., (13, 14)]. Unfortunately, it is impossible to resolve this conundrum based on the reported data.

In our opinion, it is regrettable that the usefulness of such valuable data on the issue of mind wandering and blanking in mood disorders is hampered by the conceptual vaguenesses described above. Similar problems could be avoided by precisely defining the states of consciousness under investigation, by adhering to commonly accepted classifications, and by implementing experience sampling probes closely mirroring these classifications.

## Author Contributions

All authors listed have made a substantial, direct and intellectual contribution to the work, and approved it for publication.

## Conflict of Interest

The authors declare that the research was conducted in the absence of any commercial or financial relationships that could be construed as a potential conflict of interest.
